# Clinical profile of a Polish cohort of children and young adults with cystinuria

**DOI:** 10.1080/0886022X.2020.1860089

**Published:** 2020-12-22

**Authors:** Marcin Tkaczyk, Katarzyna Gadomska-Prokop, Iga Załuska-Leśniewska, Kinga Musiał, Jan Zawadzki, Katarzyna Jobs, Tadeusz Porowski, Anna Rogowska-Kalisz, Anna Jander, Meritrafat Kirolos, Adam Haliński, Aleksandra Krzemień, Aleksandra Sobieszczańska-Droździel, Katarzyna Zachwieja, Bodo B. Beck, Przemysław Sikora, Marcin Zaniew

**Affiliations:** a Department of Pediatrics, Immunology and Nephrology, Polish Mother’s Memorial Hospital Research Institute, Łódź, Poland; b Division of Didactics in Pediatrics, Medical University of Łódź, Łódź, Poland; c Department of Nephrology, Transplantation and Hypertension, Children’s Memorial Health Institute, Warsaw, Poland; d Department of Pediatric Nephrology and Hypertension, Medical University of Gdańsk, Gdańsk, Poland; e Department of Pediatric Nephrology, Medical University of Wrocław, Wrocław, Poland; f Department of Pediatrics, Allergology and Nephrology, Military Medical Institute, Warsaw, Poland; g Department of Pediatrics and Nephrology, Medical University of Białystok, Białystok, Poland; h Department of Clinical Genetics and Pathology, University of Zielona Góra, Zielona Góra, Poland; i Department of Pediatric Nephrology, Upper-Silesian Centre for Child's Health, Katowice, Poland; j Department of Pediatric Nephrology, Medical University of Lublin, Lublin, Poland; k Department of Pediatric Nephrology, Collegium Medicum Jagiellonian University, Krakow, Poland; l Institute of Human Genetics and Center for Molecular Medicine Cologne, University of Cologne, Cologne, Germany; m Department of Pediatrics, University of Zielona Góra, Zielona Góra, Poland

**Keywords:** Children, clinical profile, cystinuria, treatment, urolithiasis

## Abstract

**Background:**

Cystinuria is an inherited disorder that results in increased excretion of cystine in the urine. It accounts for about 1–2% of pediatric kidney stones. In this study, we sought to identify the clinical characteristics of patients with cystinuria in a national cohort.

**Methods:**

This was a retrospective study involving 30 patients from the Polish Registry of Inherited Tubulopathies. Initial data and that from a 6-month follow-up were analyzed. Mutational analysis was performed by targeted Sanger sequencing and, if applicable, MLPA analysis was used to detect large rearrangements.

**Results:**

*SLC7A9* mutations were detected in 15 children (50%; 10 males, 5 females), *SLC3A1* mutations in 14 children (47%; 5 males, 9 females), and bigenic mutations in one male patient. The first clinical symptoms of the disease were detected at a median of 48 months of age (range 3–233 months). When individuals with different mutations were compared, there were no differences identified in gender, age of diagnosis, presence of UTI or urolithiasis, eGFR, calcium, or cystine excretion. The most common initial symptoms were urolithiasis in 26 patients (88%) and urinary tract infections in 4 patients (13%). Urological procedures were performed in 18 out of 30 (60%).

**Conclusions:**

The clinical course of cystinuria is similar among patients, regardless of the type of genetic mutation. Most patients require surgery before diagnosis or soon after it. Patients require combined urological and pharmacological treatment for prevention of stone recurrence and renal function preservation.

## Introduction

Cystinuria is a rare, inherited disorder that results in increased excretion of the amino acid cystine from the body. As cystine is poorly soluble in urine, excess cysteine builds up in the kidneys, which leads to the formation of recurrent kidney stones that can create blockages in the urinary tract, negatively affecting kidney function and acting as a nidus for infections [1,2]. The disease accounts for about 1–2% and up to 25% of adult and pediatric patients with kidney stones, respectively [3]. Cystinuria is an autosomal recessive disease but some heterozygous carriers have an autosomal dominant, incomplete penetrance phenotype with elevated urinary cystine excretion. These variations in cystine excretion result in broad clinical variability [4].

Cystinuria has been linked to the abnormal function of a protein transporter in the proximal convoluted tubule leading to loss of dibasic and neutral amino acids. So far, two genes responsible for cystinuria have been identified: *SLC3A1* (chromosome 2p21) encodes the heavy subunit rBAT of a renal b0,+ transporter, while *SLC7A9* (chromosome 19q12) encodes its interacting light subunit b0,+AT [5]. While these mutants also disrupt reabsorption of lysine, arginine, and ornithine, these amino acids do not form crystals when they accumulate and thus do not present clinically with stones as excess cystine does. In the absence of stone formation, cystinuria can be asymptomatic. Symptoms appear with the development of stones and can vary on a spectrum of frequency and severity. Presentation is similar to that of any other urinary calculi and includes nausea, vomiting, ‘colicky’ pain, and hematuria. Based on the severity of the disease, obstructive syndromes, recurrent urinary tract infections (UTIs), and even organ damage can occur. With the persistence of these symptoms, chronic pain is also a common complaint in patients with cystinuria [2,4,6,7].

The global prevalence of cystinuria is approximately 1 in 7000, however, this rate ranges widely in different regions (1 in 2500 neonates in Libyan Jews to 1 in 100 000 in Swedes) [1,3,4,7]. To date, there have been no reports on the prevalence, genotypes, or phenotypes of cystinuria in the Polish population. Thus, in this study we sought to elucidate the clinical characteristics of cystinuria in a national cohort of patients in Poland to facilitate improvements in detection and treatment of specific, previously unidentified subgroups of patients in this population.

## Material and methods

### Patients

This retrospective study included 30 patients (16 males and 14 females; 27 children and 3 young adults) from 26 families that had a molecular diagnosis of cystinuria and for whom data had been collected in the Polish Registry of Inherited Tubulopathies (POLtube). The patients were recruited to the registry from 9 pediatric nephrology centers throughout Poland from 2013 to 2018. The retrospective nature of the study exempted it from the Ethics Committee approval by Polish Regulation. The calculated prevalence of the disease among children in the cohort was 3.9 cases per million.

The analyses focused on clinical and biochemical data acquired upon diagnosis of cystinuria. This included patient age at initial presentation, age at clinical diagnosis, age and method of biochemical diagnosis, gender, parental consanguinity, family history of cystinuria, presenting symptoms, renal function defined as estimated glomerular filtration rate (eGFR), calciuria, 24-h cystine excretion (if available), and ultrasonography findings.

All patients were initially diagnosed based on nitroprusside testing, which is a standard test performed in the evaluation of children with recurrent stones and familial urolithiasis in Poland. Cystinuria was defined as increased excretion of cystine in the urine exceeding the normal daily excretion rate of 30 mg/day or a positive nitroprusside test result, which quantitatively assesses the excretion of cystine over 75 mg/g of creatinine. Hypercalciuria was defined by the increased excretion of calcium (>4 mg/kg/day) in the urine. UTI was diagnosed based on clinical criteria for clinical symptoms, urine testing and positive leukocyte and/or nitrite in urinary culture (if available).

Data were collected based on the initial methods used to treat cystinuria, and whether surgical interventions were required. At the time of analysis, no specific, unified protocol for pharmacological cystinuria treatment was approved in Poland. The treating physicians decided on treatment based on their knowledge and experience. Clinical and biochemical data from patients at the 6-month follow-up assessment were analyzed, when available.

A patient was diagnosed as having a recurrent stone when at least one new stone was detected by ultrasound or expulsed (provided it had been removed before). From the clinical perspective, in the follow-up period, full clinical improvement was defined as no clinical or ultrasound signs of urolithiasis at the end of observation or UTI at any point. Partial improvement was recorded as reduction of stone number. Clinical and biochemical data were later compared between 2 main groups – patients carrying a defect in the *SLC3A1* gene vs. patients with a *SLC7A9* defect.

### Genetic analysis

Mutational analysis in probands and affected family members (when available) was performed by targeted Sanger sequencing of the entire *SLC3A1* and *SLC7A9* genes, and if applicable, by using multiplex ligation-dependent probe amplification (MLPA) analysis to detect large genomic rearrangements. Appropriate approvals from parents and caregivers were obtained for genetic analysis.

### Statistical analysis

Data were recorded and analyzed using standard methods of descriptive statistics with structural measures and non-normal distribution descriptions (median value and range). Differences between groups were analyzed by the Fisher’s exact test and the Mann–Whitney two-tailed test for non-parametric data. Data management was performed with MS Excel software with detailed analyses performed with Statistica 13 PL package.

## Results

### Parameters of diagnosis

Patient characteristics are presented in Table 1. None of the patients had consanguineous parents. A family history of stone disease was reported in 13 out of 30 patients. *SLC7A9* mutations were detected in 15 patients (50%; 10 males, 5 females), *SLC3A1* mutations in 14 (47%; 5 males, 9 females), and bigenic mutations in one male patient. All patients but one had two mutated alleles. The first clinical symptoms of urolithiasis (lumbar/abdominal pain, dysuria, UTI) were detected at a median of 48 months of age (range 3–233 months). By ultrasound, 13 subjects had unilateral stones (left side – 7, right side – 6), 13 had stones on both sides. In 4 patients, UTI accompanied urolithiasis (Table 1).

**Table 1.** Clinical and genetic characteristics of patients with cystinuria from the study group.

ID	Gender	Mutated gene	Mutation	Mutation	Age of first symptoms (months)	Clinical manifestation	Age of clinical diagnosis (months)	Age of molecular diagnosis (months)	eGFR (ml/min/1.73 m^2^) at diagnosis^a^	Initial treatment	Urological treatment before diagnosis^b^	Urological treatment soon after diagnosis	eGFR (ml/min/1.73 m^2^) at follow-up	Stones in ultrasound at follow-up
F1.1	F	*SLC3A1*^c^	SLC3A1 c.1640C > T (hetero)	SLC3A1 c.424C > G (hetero)	24	UTI; UU (L)	24	24	169	IFI, DR, CIT	ESWL	ESWL	153	no
F1.2	F	*SLC3A1*	SLC3A1 c.1640C > T (hetero)	SLC3A1 c.424C > G (hetero)	72	SCR	72	72	119	IFI, DR	none	none	122	no
F2	M	*SLC7A9*	SLC7A9 c.419T > C (hetero)	SLC7A9 c.955G > A (hetero)	48	USNP	58	65	112	CIT	none	none	115	no
F3	M	*SLC7A9*	SLC7A9 c.313G > A ( homo)		3	BU	5	107	72	IFI, DR, CIT, CAP	none	DJ	83	UU (L)
F4	M	*SLC7A9*	SLC7A9: c.368C > T (hetero)	SLC7A9: c.604 + 2T > C (hetero)	60	BU, B	120	144	109	IFI, DR, CIT, CAP	ESWL (3), URS-L (2), OS	ESWL, PCNL	99	UU (L)
F5	F	*SLC7A9*	SLC7A9: c.604 + 2T > C (homo)		12	BU	12	96	101	IFI, DR, CIT, CAP	ESWL	ESWL	85	no
F6	F	*SLC7A9*^c^	SLC7A9: c.604 + 2T > C (hetero)	SLC7A9: c.694delT (hetero)	25	UTI; UU (R), B	30	72	100	IFI, DR, CIT, CAP	none	none	101	B
F7	F	*SLC3A1*	SLC3A1: c.765 + 1G > A (hetero)	SLC3A1: dup Ex5–9 (hetero)	10	BU	12	156	81	IFI, DR, CIT, CAP	OS	PCNL	111	BU
F8	F	*SLC3A1*	SLC3A1: c.1084C > T (hetero)	SLC3A1: del Ex1–5 (hetero)	48	UU (R)	48	132	125	IFI, DR, CIT, CAP	OS	PCNL	119	no
F9	M	*SLC3A1/ SLC7A9*	SLC7A9: c.313G > A (hetero)	SLC3A1: del Ex1–7 (hetero)	11	UTI; UU (L)	11	48	165	DR, TP	ESWL (5), URS-L	ESWL + PCNL	150	UU (L)
F10	F	*SLC3A1*	SLC3A1: c.845A > G (hetero)	SLC3A1: c.1094G > A (hetero)	53	BU	59	61	122	IFI, DR, CIT	none	none	109	UU (L)
F11	F	*SLC3A1*	SLC3A1: c.789T > G (hetero)	SLC3A1: c.1364C > T (hetero)	7	BU	20	72	106	IFI, DR, CIT, CAP	OS	PCNL	110	BU
F12	M	*SLC7A9*	SLC7A9: c.604 + 2T > C (hetero)	SLC7A9: c.313G > A (hetero)	3	BU	3	120	NA	IFI, DR	ESWL (6), OS (2)	ESWL + PCNL	116	no
F13	M	*SLC7A9*	SLC7A9: c.313G > A (hetero)	SLC7A9: c.368C > T (hetero)	NA	UU (L)	NA	NA	NA	NA	none	none	98	UU (L)
F14	M	*SLC7A9*^c^	SLC7A9: c.671C > T, p.Ala224Val (hetero)	SLC7A9: c.1305G > A, p.Trp435Ter (hetero)	72	UU (L)	84	180	147	IFI, DR, CIT	none	none	131	no
F15	M	*SLC7A9*	SLC7A9 c.857C > T (hetero)	SLC7A9 Deletion (hetero)	144	BU	156	157	100	IFI, DR, CIT	ESWL	ESWL	101	UU (R)
F16	M	*SLC3A1*	SLC3A1: c.2019C > A, p.Cys673Ter (homo)		207	UU(R)	208	NA	82	IFI, DR, CIT	none	none	88	UU (R)
F17	F	*SLC3A1*	SLC3A1: c.1354C > T (homo)		6	UU (R) staghorn	7	7	NA	IFI, DR, CIT, TH	PCNL	ESWL (2)	120	no
F18	F	*SLC7A9*	SLC7A9 c.1060G > A (hetero)	SLC7A9 c.604 + 2T > C (hetero)	144	BU	291	293	148	IFI, DR, CIT	none	none	124	BU
F19	M	*SLC3A1*	SLC3A1 c.256C > T (hetero)	SLC3A1 c.1796T > C (hetero)	14	BU	88	94	93	NA	ESWL	ESWL	90	UU (L)
F20	M	*SLC3A1*	SLC3A1:c.1069_1084dup (hetero)	SLC3A1: dup Ex5–9 (hetero)	133	UU (L)	150	153	73	IFI	none	none	92	UU(L)
F21	M	*SLC7A9*^c^	SLC7A9: c.1252A > C, p.Thr418Pro (hetero)	SLC7A9: c.1445C > T, p.Pro482Leu (hetero)	11	UTI; UU (L)	12	132	90	IFI, DR, CIT, CAP	URS-L (2)	TULT	84	no
F22.1	M	*SLC7A9*^c^	SLC7A9: c.422A > G (hetero)	SLC7A9: c.583G > A (hetero)	144	BU	144	216	96	IFI, DR, herbs	none	None	95	UU (R)
F22.2	F	*SLC7A9*	SLC7A9: c.422A > G (hetero)	SLC7A9: c.583G > A (hetero)	204	UU (L)	207	198	93	IFI, DR, CIT	OS	PCNL	99	UU (L)
F23.1	F	*SLC7A9*	SLC7A9:c.604 + 2T > C (hetero)	SLC7A9:c.955G > A, p.Gly319Arg (hetero)	233	BU	301	302	113	IFI, DR, CIT, CAP	ESWL, RIRS	none	112	no
F23.2	M	*SLC7A9*	SLC7A9:c.604 + 2T > C (hetero)	SLC7A9:c.955G > A, p.Gly319Arg (hetero)	NA	SCR	202	205	86	IFI, DR, CIT, CAP	none	none	96	no
F24	F	*SLC3A1*^d^	SLC3A1:c.17G > A (p.Ser6Asn) (hetero)		132	UU (R)	149	149	120	IFI, DR, CIT, TP	URS-L	URS-L	118	UU (R)
F25	M	*SLC3A1*	SLC3A1:c.1400T > C (p.Met467Thr) (homo)		NA	BU	288	288	93	IFI, DR, CIT, TP	URS-L, ESWL, RIRS, PCNL	none	100	UU (R)
F26.1	F	*SLC3A1*	SLC3A1: Duplikation Exons 5–9 (hom)		18	UU (R)	92	278	105	DR, CIT	ESWL	none	115	UU (R)
F26.2	M	*SLC3A1*	Duplikation Exons 5–9 (hom)		42	SCR	43	204	135	DR, CIT, CAP	none	ESWL	106	no
median	–	–	**–**	–	48	–	72	138	105	–	–	–	108	–
range	–	–	–	–	3–233	–	3–301	7–302	72–169	–	–	–	83–153	–

Abbreviations: B – urinary bladder; BU – bilateral urolithiasis; CAP– captopril; CIT – potassium citrates; DR – dietary restrictions; eGFR – estimated glomerular filtration rate; ESWL – extracorporeal shockwave lithotrypsy; F– female; IFI – increased fluid intake; M – Male; NA – not available; OS – open surgery; PCNL – percutaneous nephrolitotomy; RIRS – retrograde intrarenal surgery; SCR – screening in symptomless child; TP – tiopronin; TULT – transureteral litotomy, UNSP – unspecific abdominal pain; URS-L – ureterorenoscopy-lithotrypsy; UTI – urinary tract infection; UU – unilateral urolithiasis (L-left, R-right).

^a^Glomerular filtration rate was estimated in adults using the Modification of Diet in Renal Disease criteria, and for patient <18 years using the modified Schwartz formula (*k* = 0.413).

^b^Number of urological interventions (if more than one) in brackets.

^c^Novel mutation.

^d^Heterozygous mutation.

Diagnosis of cystinuria was established at a median age of 72 months (range 3–301 months). The median delay between first presentation of the disease and clinical diagnosis of cystinuria was 10 months (range 0–288). In 3 patients, diagnosis was established in early adulthood. The age of diagnosis was similar for *SLC7A9* and *SLC3A1* mutation carriers (median 60 vs. 42 months, respectively; *p* = 0.12). In 4 children (13%), diagnosis was established by screening the families of the probands or by the parents’ decision.

Initial diagnosis of cystinuria was based on both clinical symptoms (stone presence in ultrasound, UTI/screening in families – Table 1) and biochemical analysis of urine by nitroprusside screening (most patients), or by 24-h urine excretion of cystine (5 pts) or stone analysis (3 pts) together. Before the initiation of any treatment, 24-h cystine excretion was assessed in 18 patients (60%), which ranged from 52 to 1545 mg/g with a median of 469 mg/g of creatinine. Hypercalciuria was present in 2 patients. Genetic confirmation of cystinuria was determined at a median age of 138 months (range 7–302 months) with a median time of 46 months from the clinical diagnosis date.

One patient presented with acute renal injury with a maximal eGFR decrease to 34 mL/min/1.73 m^2^ due to post-renal failure (as a result of bilateral ureteral blockage and bladder stones). Data on renal function at the time of cystinuria diagnosis were available for 27 patients. Their median eGFR was 105 (range 72–169) mL/min/1.73 m^2^. Five patients (18%) were found to have slightly impaired eGFR (range 72–86 mL/min/1.73 m^2^; children < 2 years of age were excluded).

When comparing individuals with mutations in *SLC3A1* versus *SLC7A9*, there were no differences in gender (*p* = 0.37), age of presentation (*p* = 0.07), age of clinical diagnosis (*p* = 0.12), presence of UTI (*p* = 0.45) or urolithiasis (*p* = 0.57), eGFR (*p* = 0.11), calcium (*p* = 0.22), or cystine excretion (*p* = 0.07) (Table 2). There was no effect of gender on the above parameters.

**Table 2.** Basic clinical and biochemical differences between patients with *SLC3A1* and *SLC7A9* mutations at the clinical diagnosis.

	Gender F:M ratio	Age of first symptoms (months)	UTI presence	Age of clinical diagnosis (months)	Age of molecular diagnosis (months)	Stone presence	eGFR (ml/min/1.73 m^2^) at diagnosis	Cystine excretion (mg/24 h)	Calcium excretion mg/kg/24 h
SLC3A1 (*n* = 14)	9:5	42 (6–207)	2/14	65 (7–288)	132 (7–288)	14/14	116 (78–175)	317 (52–1127)	2.4 (1.0–4.9)
SLC7A9 (*n* = 15)	5:10	60 (3–233)	3/15	102 (3–301)	150 (65–302)	12/15	128 (61–175)	680 (271–1545)	2.3 (0.2–4.9)
Statistical difference (p)	0.08	0.12	0.45	0.09	0.56	0.57	0.11	0.07	0.22

One patient with bigenic mutation was excluded. Data presented as a median (range).

### Treatment

Data on treatment were available for 28 patients (Table 1). The majority (89%) had their fluid intake increased after a clinical diagnosis was made, which is recommended as a standard prevention for stone formation in cystinuria. In 3 patients (10.7%), no dietary restrictions (i.e., a low salt diet and reduced protein intake) were advised. Among pharmacological treatments, potassium citrate was the most commonly prescribed (in 24 patients; 85.7%). Captopril and tiopronin were given to 10 (35.7%) and 4 (14.3%) patients, respectively. Standard initial potassium citrate dosage was 0.5 mEq/kg/day. Parents were instructed to adjust dosage to maintain a high urine pH of 7.7–8.0 at a final dose of 1–1.1 mEq/kg/day. Captoprilum was given at a dosage of 0.5–1.0 mg/kg/day. Triopronin was administered with an initial dose of 15 mg/kg/day dose and finally ranged 300–900 mg (5–30 mg/kg/day).

### Urological interventions

Prior to the establishment of a diagnosis, multiple types of urological interventions were performed to remove stones in 18 out of 30 patients (60%) (Table 1). When on pharmacological treatment, 16 out of the 30 patients still required urological interventions (53%) after 6 months. The procedures mostly involved preexisting stones and were performed soon (1–8 weeks) after diagnosis because new stone formation was detected in a significantly lower number of subjects (described below).

### 6-Month follow-up

6-Month follow up revealed that 7 out of 30 patients (23%) had recurrent stones (one required new surgery-PCNL) and 2 out of 30 patients (7%) had a UTI. At the end of the follow-up period, renal function was mostly normal (median 109; range 84–153 mL/min/1.73m^2^). Only 2 out of 5 children with previously impaired renal function did not completely recover but other than stone complications, no other risk factors were reported. Daily urine production, which reflects fluid intake, increased to a median value of 1800 mL (range 380–4800 mL) from 1400 mL (range 370–4500 mL). From a clinical point of view, full clinical improvement (free of stones) after 6 months was reported in 12 patients (40%) and partial improvement in 6 patients (20%) while 12 (40%) patients still had stones. When comparing patients who had new recurrent urolithiasis in follow-ups with those who did not, no significant difference was found in terms of the type of mutation, age of first symptoms and age at clinical diagnosis, presence of UTI or urolithiasis at initial diagnosis, cystine excretion, or eGFR. The only significant difference was found for the initial 24-h urine calcium excretion, which was lower in those who produced no stones when compared to those who did (mean 1.62 vs. 3.8 mg/kg/24h; *p* = 0.024) – Table 3.

**Table 3.** Basic clinical and biochemical differences between patients with no new/recurrent stones and still producing stones after the clinical diagnosis.

	Gender F:M ratio	*SLC3A1/ SLC7A9*^a^	Age of first symptoms (months)	Age of clinical diagnosis (months)	UTI presence	eGFR (ml/min/1.73 m^2^) at diagnosis	Initial cystine excretion (mg/24 h)	Follow-up cystine excretion (mg/24 h)	Initial calcium excretion (mg/kg/24 h)	Follow-up calcium excretion (mg/kg/24 h)
Successful treatment (*n* = 18)	8/10	8/9	36 (3–233)	86 (3–301)	3/18	100 (61–186)	660 (52–1545)	1360 (260–2600)	1.62 (0.2–2.9)	2.1 (0.2–8.1)
Stone still present (*n* = 12)	6/6	5/6	65 (3–204)	51 (5–281)	2/12	118 (72–165)	1100 (209–1200)	1212 (210–3060)	3.8 (2.1–4.9)	1.97 (1.8–6.4)
Statistical difference (*p*)	0.32	0.34	0.63	0.97	0.12	0.37	0.09	0.73	0.024	0.45

Data presented as a median (range).

^a^In 29 patients (bigenic mutation excluded).

## Discussion

We present here for the first time, characteristics of a national cohort of patients with cystinuria in the Polish population. The results presented in this study were made possible through the invaluable help of the POLtube Registry, which reviews the collection of blood samples and clinical data from patients with various tubulopathies, and provides access to genetic testing, facilitating molecular diagnosis and genetic counseling. Here we provide data on genetic defects in a large, national cohort of patients with cystinuria. When compared to other national data, the number of patients appears low, which suggests an underestimation of cystinuric patients in Poland. Despite this, coverage of most parts of Poland was maintained and thus the cohort is representative of the whole country, ensuring that the results obtained were not biased. In addition, the number of observations from the clinical national data is comparable to those reported in previous studies from Korea, Portugal, France [8], and Great Britain [9–12]. All clinical pediatric research centers dealing with tubulopathies in Poland were engaged in the study, making the clinical analysis of pediatric cystinuria patients the first and largest of its kind in Poland. The prevalence calculated in our study (0.39 in 100 000) was significantly lower than expected in European populations (1 in 100 000). Thus, we assumed that this was an underestimation of the actual number of cystinuric patients in the country.

Genotypic analysis of the Polish cohort showed equal distribution of *SLC3A1* and *SLC7A9* mutations. Of note, we did not find any effect of the respective gene defects on the analyzed parameters at diagnosis, or during the clinical course of the disease. This is in line with other studies including one by Rhodes et al., which found that the mutations in the *SLC3A1* and *SLC7A9* genes in cystinuria patients resulted in indistinguishable disease manifestations [8,13]. Similarly, Wong et al. found no significant differences in multiple clinical parameters, such as age at disease presentation or number of urolithiasis episodes when comparing groups of patients with different genotypes [10]. Similar data were reported for Korea, Czechia, Slovakia, and Greece [9,14].

One of the most important findings we revealed in this study was delay in cystinuria diagnosis. Notably, a significant proportion of patients remained undiagnosed for a long time despite presenting with the suggestive clinical symptoms, including recurrence of stones or bilateral renal localization (13/30; 43% patients – Table 1). The delay in diagnosis could possibly be due to the rarity of the disease, and an insufficient awareness is also likely a contributing factor. Although clinical guidelines recommend that an exhaustive metabolic evaluation, including urinary cystine analysis, should be conducted in cases of recurrent stone disease and/or diagnosis of urolithiasis at an early age, this is not typically performed [1,2]. The delay in diagnosis, along with the patient number in this study compared to others in the literature, suggests that cystinuria is likely underdiagnosed and/or underreported in Poland. In this respect, much work must be done to improve identification of the disease. Interestingly, urine nitroprusside screening still remains a standard test in most pediatric nephrology centers. However, it should be noted that this method is considered outdated and is not available in every laboratory. Therefore, a quantitative assessment of cystine in the urine is the recommended method [2]. The underutilization of this method may also explain the lack of timely diagnosis. As far as we know, there is restricted access to urine cystine assessment in Poland. Correctly diagnosing cystinuria has great clinical importance, such as reducing urological procedures, as was the case with in our patient cohort. Urological interventions were performed in more than half of our patients before diagnosis, which could possibly be avoided in some cases.

Chronic inflammation and urine blockage have been described as risk factors for permanent renal injury. Data from Brazil showed significant scarring in 13 (69%) pediatric patients with cystinuria, who had a follow-up in a single tertiary institution between 2004 and 2016. However, mean eGFR was not decreased at 92–106 mL/min/1.73 m^2^ [15]. In a large study of adolescents and adults with cystinuria, a significant increase in serum creatinine was detected in cystinuric patients when compared to calcium oxalate stone formers [16]. In our study, renal function was mostly preserved with slightly decreased eGFR in 5 patients (4 with a slight decrease and one with significant acute renal injury) at time of diagnosis. Successful combined (surgical and pharmacological) therapy restored impaired renal function in 28 out of 30 patients after a 6-month observation. Reasons for impaired eGFR in the patients varied. One had an acute bilateral blockage (as mentioned above), while the remaining 4 had slightly lowered eGFR (above 80 mL/min/1.73 m^2^). In our opinion, this might be the result of chronic changes in the renal parenchyma due to recurrent urolithiasis, with a component of subclinical unilateral obstruction with UTI – this was present in only 2 patients after follow-up. Unfortunately, no data from imaging studies (scintigraphy, CT) were available to support this hypothesis. Most patients required surgery before clinical diagnosis, which is typical of cystinuria. However, even after introducing pharmacological therapy, the majority of children were operated on. On the other hand, in 17 out of 18 cases, surgery was required due to the formation of stones before pharmacological treatment, so we can postulate the need for surgery was in fact reduced [11,15,16].

At the time of diagnosis, the vast majority of patients presented with urolithiasis and a significant number with UTIs. These symptoms appear to have improved after initiation of treatment, with 60% reporting a reduction in stones and a decrease in the need for surgery at the 6-month follow-up (only 1 patient needed new surgery). This suggests that the current recommendations for disease management are beneficial for clinical improvement, particularly soon after initiation of treatment. For those with partial or no improvement and continue to have recurrent symptoms, alternate treatment options might be further explored [2,17]. The medical management of patients in this study included increasing the rate of diuresis, alkalization of the urine with potassium citrate to promote cystine solubility, and addition of cysteine-binding thiols, as per management guidelines used worldwide [2,4,17]. In this regard, while the given therapy was effective in a significant number of patients, we feel it was not optimal. Not all patients were advised on increased fluid intake and diet restrictions, and citrates were not prescribed to all patients. Nevertheless, fluid intake was not satisfactory, though diuresis (which reflects intake) increased on observation. Furthermore, a significant number of patients received captopril, which had been advised earlier [1]. At present, tiopronin should be used instead. Unfortunately, tiopronin was only given to 4 patients. We are sure, that it was not the optimal choice. On the other hand, this observation is in line with the therapeutic algorithm proposed by Barbey et al., which described thiols as a second-line therapy that should only be used when basic measures fail to control cystine stone formation [17]. This may be explained by physicians being hesitant to prescribe tiopronin, as it is an expensive medication and not freely available in Poland. Because of the retrospective design of the study, we had only gathered information from participating centers. There were no published nationwide guidelines on how to treat cystinuria. Therefore, the treating physicians decided on treatment based on their knowledge and experience. The non-optimal strategy of cystinuria treatment in Poland is one of the important conclusions of the study. Unfortunately, the retrospective design of the study and a short observational time limit any conclusions on the long-term (years) effectiveness of treatment.

## Conclusions

In this study, we analyzed the clinical manifestations of cystinuria in a cohort of 30 pediatric patients throughout Poland and found no significant differences in clinical manifestations among the patients, regardless of which genetic mutation was detected, the patients’ gender, or the time of diagnosis.

Most cystinuric children required surgery before diagnosis, and soon after diagnosis. The patients require combined urological and pharmacological treatment for prevention of stone recurrence and renal function preservation. Pharmacological treatment was mostly effective but did not provide full prevention of stone recurrence or a need for surgery within 6 months of clinical diagnosis. However, prognosis in terms of renal function was good.

Our study suggests that the disease may be underdiagnosed in Poland, which requires action such as educational interventions regarding symptoms and diagnosis methods.
